# Systems and Evolutionary Characterization of MicroRNAs and Their Underlying Regulatory Networks in Soybean Cotyledons

**DOI:** 10.1371/journal.pone.0086153

**Published:** 2014-01-27

**Authors:** Wolfgang Goettel, Zongrang Liu, Jing Xia, Weixiong Zhang, Patrick X. Zhao, Yong-Qiang (Charles) An

**Affiliations:** 1 United States Department of Agriculture, Agricultural Research Service, Plant Genetics Research Unit, Donald Danforth Plant Science Center, Saint Louis, Missouri, United States of America; 2 United States Department of Agriculture, Agricultural Research Service, Appalachian Fruit Research Station, Kearneysville, West Virginia, United States of America; 3 Department of Computer Science and Engineering, Washington University, Saint Louis, Missouri, United States of America; 4 Plant Biology Division, The Samuel Roberts Noble Foundation, Ardmore, Oklahoma, United States of America; National Institutes of Health, United States of America

## Abstract

MicroRNAs (miRNAs) are an emerging class of small RNAs regulating a wide range of biological processes. Soybean cotyledons evolved as sink tissues to synthesize and store seed reserves which directly affect soybean seed yield and quality. However, little is known about miRNAs and their regulatory networks in soybean cotyledons. We sequenced 292 million small RNA reads expressed in soybean cotyledons, and discovered 130 novel miRNA genes and 72 novel miRNA families. The cotyledon miRNAs arose at various stages of land plant evolution. Evolutionary analysis of the miRNA genes in duplicated genome segments from the recent *Glycine* whole genome duplication revealed that the majority of novel soybean cotyledon miRNAs were young, and likely arose after the duplication event 13 million years ago. We revealed the evolutionary pathway of a soybean cotyledon miRNA family (soy-miR15/49) that evolved from a neutral invertase gene through an inverted duplication and a series of DNA amplification and deletion events. A total of 304 miRNA genes were expressed in soybean cotyledons. The miRNAs were predicted to target 1910 genes, and form complex miRNA networks regulating a wide range of biological pathways in cotyledons. The comprehensive characterization of the miRNAs and their underlying regulatory networks at gene, pathway and system levels provides a foundation for further studies of miRNAs in cotyledons.

## Introduction

MicroRNAs (miRNAs) are a class of ∼21 nt long non-coding RNAs that repress expression of their targeted genes in eukaryotes, predominantly at the post-transcriptional level. Canonical miRNAs are produced from their own miRNA genes (*MIR*), which are mostly transcribed by RNA polymerase II (Pol II) [1], [2]. The resulting single-stranded primary miRNAs (pri-miRNAs) then fold into stem-loop structures that are recognized by the RNase III type enzymes Dicer-like (DCL). DCL proteins (mostly DCL1) typically cleave first at the base of the stems generating the miRNA precursor hairpins (pre-miRNAs) and then at the loop regions of the precursors to liberate the miRNA/miRNA* duplexes from the hairpins [3]–[5]. The 3′ overhangs of the duplex that result from the staggered dicing activity are methylated by Hua Enhancer 1 (HEN1) to protect the duplex from degradation. The mature miRNAs are incorporated into Argonaute (AGO) family proteins, mostly AGO1 and the RISC effector complex, that target mRNAs for slicing through perfect or partially complementary base pairing [6]. miRNA* are generally considered as by-product or non-functional [3]–[5], but a few miRNA* are functional and can interact with AGO proteins to exert their function [7]. In plants, most miRNAs perfectly or nearly perfectly match coding regions or 5′ and 3′ UTRs of their mRNA targets resulting in transcript cleavage [8]–[11]. miRNAs may also imperfectly bind to 3′ UTRs of target genes to cause translational inhibition [11], [12]. miRNAs, especially 22nt long miRNA species, are also able to trigger phased RNA (PhasiRNA)/trans-acting siRNA (tasiRNA) production in both non-coding (e.g. TAS genes) and coding gene loci [13], [14].

Plant miRNAs are known to regulate diverse biological processes, including development, organ identity, metabolism, and stress response [15]–[18]. Although plant miRNAs regulate genes with diverse biological functions, they preferentially target transcription factor genes, suggesting that miRNAs are at key positions in gene regulatory networks underlying those biological processes. Some miRNAs and their targets are evolutionarily conserved across many orders of divergent plant species while others are young and species-specific. Conserved miRNAs tend to have large families with well-defined targets and accumulate at high levels. In contrast, non-conserved and young miRNAs tend to have small families with less-defined targets and accumulate at low levels [19]–[22]. Young miRNAs are often excised less precisely, and their length deviates more from the typical 21nt size compared to ancient and conserved miRNAs [20]. Accumulation of miRNAs is often differentially regulated with respect to specific tissues, developmental stages and environmental signals, and may differ by several orders of magnitude [23]–[26]. It often requires high-depth sequencing of small RNAs to identify young miRNAs in any given tissue.

Soybean is an important dual-purpose crop, which provides both seed protein and oil for animal feed and human consumption. Soybean cotyledons evolved as specialized sink tissue for the synthesis and storage of protein and oil. Soybean is a paleopolyploid with a genome of approximately 1.1 gigabases [27]. Its genome went through two rounds of whole genome duplications occurring at approximately 59 and 13 million years ago, which resulted in a highly duplicated genome with nearly 75% of its genes present in multiple copies [27]. The highly duplicated genome and the dynamic genome rearrangements provide opportunities for the evolution of novel miRNAs.

Computational prediction and sequencing of small RNAs have been used to discover miRNAs in a number of soybean tissues [28]–[34]. To gain insight into both evolution of miRNAs and their underlying regulatory networks in soybean cotyledons, we carried out an extensive and comprehensive characterization of miRNAs expressed in soybean cotyledons. Eighteen small RNA libraries from cotyledons at various stages were sequenced to generate a total of 292 million small RNAs, which represents one of the largest collections of small RNA reads in a single tissue type in plants. The high-depth sequencing project led us to discover 130 cotyledon miRNAs and 72 cotyledon miRNA families, which have not been reported previously. The majority of soybean cotyledon miRNAs arose after the *Glycine* whole genome duplication event 13 million years ago. We showed that a novel cotyledon miRNA family recently arose through an inverted duplication of a protein-coding gene fragment. Further, we depicted its evolutionary pathway and provided strong evidence for the hypothesis that miRNAs can originate from inverted duplications of their target genes. Cotyledon miRNAs regulate a large number of genes and biological pathways with diverse biological functions, suggesting that they play central roles in gene regulatory networks in cotyledons. The regulatory networks underlying interactions of cotyledon miRNAs with their targeted genes and biological pathways were inferred to reveal their regulatory functions in soybean cotyledons.

## Results and Discussion

### Complex Composition of small RNAs in Soybean Cotyledons

A total of 18 small RNA libraries constructed with 17–30nt RNAs from cotyledons at various stages were sequenced. A total of 292 million raw sequencing reads were generated. Reads without 3′ sequencing adaptors, with ambiguous sequences or shorter than 17nt, which accounted for 13% of the raw reads, were removed from further analysis. A total of 253 million reads accounting for 87% of the total raw sequence reads were considered as qualified reads, and used for miRNA prediction and characterization. To our knowledge, these small RNA reads represent one of the largest collections of small RNA reads generated from a single tissue type in plants to date. The high-depth sequencing of small RNAs in cotyledons greatly increases the chances to identify rare and low abundant miRNAs. Eighty-five percent of the qualified small RNA reads could be mapped to the soybean genome and transcript sequences with no mismatches while the remainders were excluded from this analysis. In total, 32, 23, and 26% of the small RNAs could be perfectly mapped to protein-coding transcript, rRNA and repeated sequences, respectively. All miRNAs identified previously and in this work accounted for 24% of the small RNA population. Thus, miRNAs only contributed a small portion to small RNAs accumulated in soybean cotyledons.

**Figure 1:**
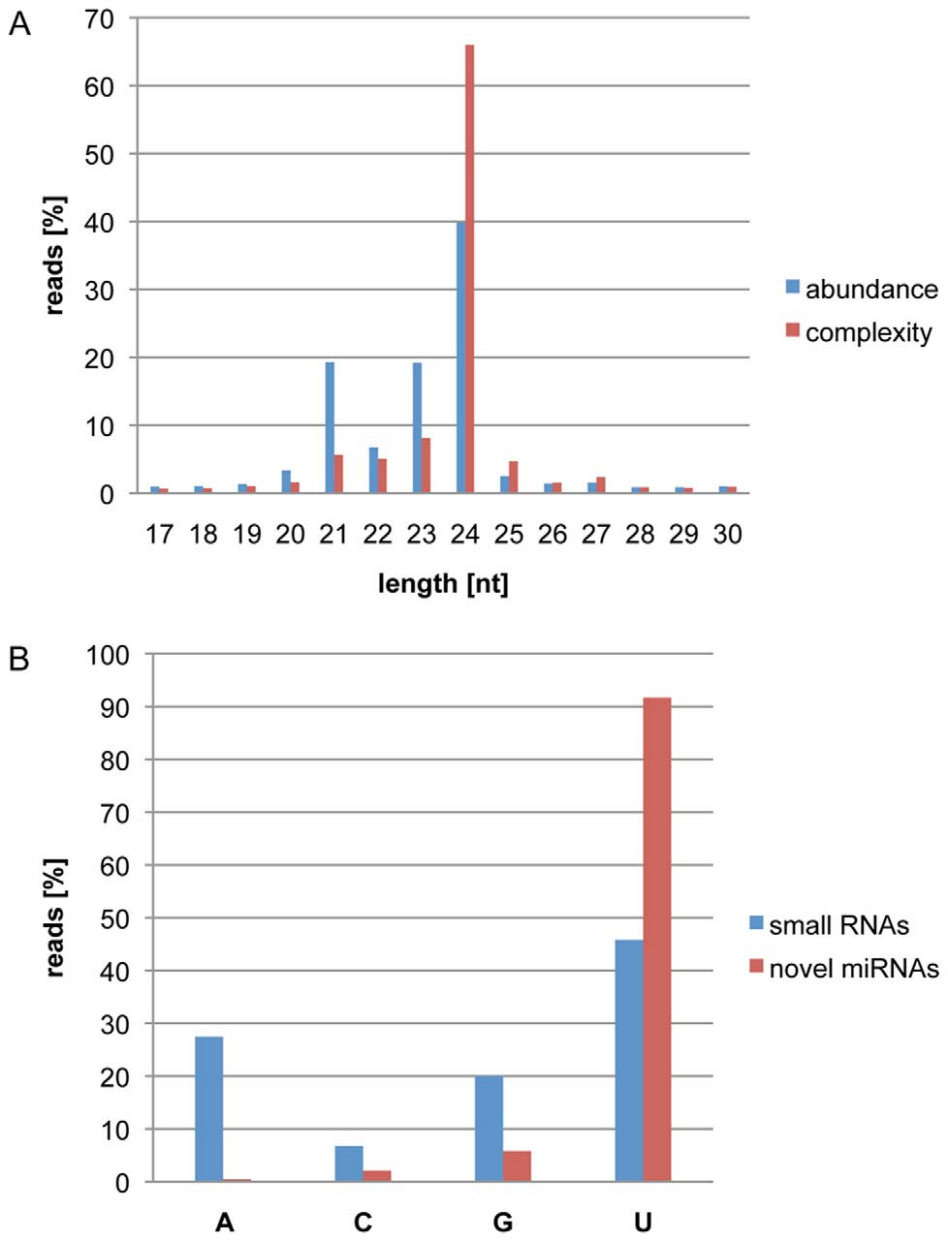
Characterization of Small RNAs in Soybean Cotyledons. (A) Abundance and complexity distribution of small RNAs at each size in soybean cotyledons: The percentages of 253 million qualified small RNA reads at each given size (abundance, in blue) and the percentages of 41 million unique small RNA species at each given size (complexity, in red) are indicated on the Y-axis. Sizes of the small RNA reads are displayed on the X-axis. (B) Highly biased presence of U at 5′ ends of newly discovered cotyledon miRNAs: The percentages of 253 million qualified reads for each nucleotide at their 5′ end (blue column) and the percentages of newly discovered miRNA reads for each nucleotide at their 5′ end in cotyledons (red column) are shown on the Y-axis. The X-axis denotes nucleotides at the 5′ end of small RNAs or newly discovered miRNAs.

The sequencing data revealed that 21, 22, 23 and 24nt RNAs were the predominant small RNAs in cotyledons, accounting for 85% of small RNA reads. The 24nt RNAs were the most abundant small RNAs, and represented 40% of all small RNA reads. A total of 41 million unique small RNA species were identified in the 253 million qualified reads, indicative of an extreme complexity in the small RNA population of cotyledons. Twenty-four nucleotide long RNAs had the highest complexity, which made up 66% of the small RNA complexity (Figure 1A), but had the lowest accumulation levels in cotyledons with 2 counts per small RNA on average (Figure S1). They most likely represent heterochromatic siRNAs [35]. In contrast, 21nt long RNAs had the highest accumulation level per small RNA species at 13 counts per small RNA on average (Figure S1), but only accounted for 6% of small RNA complexity. The extreme complexity of the small RNA population with a large percentage of low-abundant small RNAs in cotyledons suggests that high-depth sequencing and systems characterization in a plant tissue are required to gain comprehensive insight into complex small RNA networks. Small RNA compositions in diverse plant tissues and species vary greatly. Like in soybean cotyledons, it was observed that 24nt RNAs are the predominant size of small RNAs in soybean roots, nodules, flowers and developing seeds [30], [31]. However, Subramanian *et al.* showed that 20nt long RNAs are the most abundant in soybean nodules [29]. Lelandais-Briere *et al.* showed that 24nt RNAs are the most abundant in *Medicago truncatula* nodules while 21nt RNAs are the most abundant in root apex. The set of 21nt RNAs are around threefold more abundant in root tips than in nodules. Interestingly, the expression levels of MtDCL1 and MtDCL3 homologs in these tissues, which are expected to produce essentially 21 and 24nt RNAs, respectively, correlate with their accumulation levels, suggesting that the composition of small RNAs are mediated through differential expression of DCL1 and DCL3 activities [36]. It remains to be determined if higher accumulation of 24nt small RNAs were attributed by a higher DCL3 activity in cotyledon tissue.

### A Small Portion of Known miRNA Genes Were Expressed in Cotyledons

To identify the known miRNAs that accumulate in cotyledons, we compared qualified small RNA reads with the mature soybean miRNA sequences in miRBase release 20. We revealed that 174 known soybean miRNA genes representing 60 soybean miRNA families were expressed in cotyledons (Table 1 and Table S1 in File S1). At least 10 reads of a known miRNA had to be detected from our collection of small RNAs to increase the confidence of the miRNA assignment. A total of 530 individual miRNA genes and 219 miRNA families have been identified in soybean (based on miRBase release 20) [34]. Thus, only 33% of known soybean miRNAs and 27% of known soybean miRNA families were expressed in soybean cotyledons. It may reflect that cotyledons evolved into a distinct biological network system highly specialized to synthesize and deposit seed storage reserves. One hundred forty of the 174 known miRNA genes were conserved in other species and belonged to 31 miRNA families (Table 1 and Table S1 in File S1). The remaining 34 non-conserved soybean-specific miRNA genes belonged to 30 distinct families.

**Table 1:** Summary of Cotyledon miRNA Genes and Families.

	Conserved	Non-conserved	Total
No. of cotyledon miRNA genes	178	126	304
No. of cotyledon miRNA families	35	113	148
Median miRNA family size	4	1	
Median miRNA expression	2518	246	
Median precision rate (%)	87.39	63.49	
No. of known miRNA genes	140	34	174
No. of novel miRNA genes	38	92	130
No. of known miRNA families	35	41	76
No. of novel miRNA families	0	72	72

There is a significant number of miRNA genes that produce equivalent accumulation levels of miRNA and miRNA* and are named as miRNA-3P and -5P in miRBase release 20 [34]. Thirty-one of those miRNA genes were expressed in cotyledon tissue. Table 2 lists read counts for each pair of miRNA sequences in cotyledons. In our data set, only 6 of the 31 miRNA genes produced miRNA-3P and -5P reads with less than 2 fold differences in their accumulation levels. Twenty-two miRNA genes produced miRNA-3P and -5P reads with 2.1 to 2788 fold differences at their accumulation. The remaining 3 miRNA genes, *GMA-MIR171I*, *390B* and *4397* had no detectable read counts for one of the two forms in our large collection of small RNA reads. Thus, most of the miRNA-3P and -5P accumulated in cotyledon tissue at highly biased levels. We assigned more abundant small RNA sequence reads as mature miRNAs. For example, there were 1088 small RNA read counts for gma-miR394a-5P, while we only detected 1 read for gma-miR394a-3P from our collection of small RNA reads. Consequently, the miRNA-5P likely represents the mature miRNA while the miRNA-3P is a minor miRNA or a miRNA* in soybean cotyledons. Mature miRNAs are normally more abundant than miRNAs* and are incorporated into the RISC effector complex that targets mRNAs for slicing through perfect or partially complementary base pairing [3]–[6]. However, there are also cases where miRNA* strands reside in AGO1 complexes and possess gene-regulatory activity [37]. Therefore, miRNA* strands have fates other than default degradation. It has also been demonstrated that miRNA* can be preferentially sorted into AGO2 complexes in Drosophila [7].

**Table 2:** Ratio of miRNA and miRNA* for *MIRs* with Both miRNA-3P and 5P.

miRNA in cotyledon	miRNA read count	miRNA* in cotyledon	miRNA* read count	Ratio of miRNA/miRNA*
gma-miR160a-5p	30656	gma-miR160a-3p	1688	18.16
gma-miR166a-3p	234260	gma-miR166a-5p	157794	1.48
gma-miR172b-3p	499	gma-miR172b-5p	42	11.88
gma-miR396a-5p	1287	gma-miR396a-3p	93	13.84
gma-miR396b-5p	7621	gma-miR396b-3p	795	9.59
gma-miR390a-5p	4190	gma-miR390a-3p	2330	1.80
gma-miR390b-5p	3476	gma-miR390b-3p	0	
gma-miR1510b-3p	980289	gma-miR1510b-5p	28181	34.79
gma-miR482b-3p	470351	gma-miR482b-5p	18650	25.22
gma-miR4397-5p	126	gma-miR4397-3p	0	
gma-miR394b-5p	1088	gma-miR394b-3p	2	544.00
gma-miR4412-5p	37449	gma-miR4412-3p	23	1,628.22
gma-miR394a-5p	1088	gma-miR394a-3p	1	1,088.00
gma-miR4415a-3p	1625	gma-miR4415a-5p	62	26.21
gma-miR166c-3p	234260	gma-miR166c-5p	157794	1.48
gma-miR171i-3p	457	gma-miR171i-5p	0	
gma-miR5371-5p	24	gma-miR5371-3p	5	4.80
gma-miR171j-3p	2941	gma-miR171j-5p	2026	1.45
gma-miR397b-5p	2618	gma-miR397b-3p	307	8.53
gma-miR408a-3p	1123	gma-miR408a-5p	73	15.38
gma-miR408b-3p	1123	gma-miR408b-5p	33	34.03
gma-miR408c-3p	1123	gma-miR408c-5p	73	15.38
gma-miR159e-3p	16954526	gma-miR159e-5p	10467	1,619.81
gma-miR166i-3p	234260	gma-miR166i-5p	84	2,788.81
gma-miR169j-5p	163	gma-miR169j-3p	91	1.79
gma-miR169l-5p	53	gma-miR169l-3p	1	53.00
gma-miR171k-5p	346	gma-miR171k-3p	93	3.72
gma-miR172h-3p	499	gma-miR172h-5p	35	14.26
gma-miR172i-5p	35	gma-miR172i-3p	17	2.06
gma-miR396i-3p	2369	gma-miR396i-5p	1287	1.84
gma-miR482d-3p	470351	gma-miR482d-5p	18650	25.22

### A Large Number of Novel miRNA Genes Were Expressed in Cotyledons

Stringent criteria were applied to identify novel miRNA genes expressed in cotyledons, principally following the standards proposed by Meyers *et al.* [38]. We determined the miRNA processing precision rates for each predicted hairpin structure and removed miRNAs with a low precision rate. Each newly identified miRNA had to be detected in multiple small RNA libraries with a minimum of 10 reads in total, which was the identical cut-off used in identifying known miRNAs. We identified a total of 130 novel miRNA genes (Table 1 and Table S2 in File S1). The newly identified miRNAs have an obvious bias for uracil (U) at their 5′ ends. Ninety-two percent of the novel cotyledon miRNAs had a 5′ terminal uracil, while only 46% of all qualified small RNA reads had a uracil at their 5′ ends (Figure 1B). It has been shown in Arabidopsis that AGO1 proteins have a higher affinity to miRNAs containing a uracil in their first position, which could result in the enrichment of mature miRNA sequences with uracil at their 5′ termini [39], [40]. A highly biased presence of 5′ terminal uracils in miRNAs was also reported in other plant species such as maize and Medicago [36], [41], supporting the high effectiveness in our identification of bona fide soybean miRNAs.

Thirty-eight novel soybean miRNA genes belong to 20 previously identified conserved miRNA families. In addition, 13 novel miRNAs belonged to non-conserved miRNA families only identified previously in soybean. Seven miRNAs, soy-*MIR15A-F* genes and soy-*MIR49* genes, were grouped as a new family, soy-miRNA15/49 (see below). Two miRNAs, soy-miR245a and b were grouped in a new family soy-miR245. The remaining 70 *MIR* loci had mature miRNA sequences distinct from one another and from known mature miRNA sequences. Each of these miRNAs should represent a novel miRNA family with a recent origin. Thus, 130 novel soybean miRNA genes and 72 novel miRNA families were discovered and added to the current collection of soybean miRNA genes (Table 1). Together with previously identified miRNA genes, a total of 304 miRNA genes were expressed in cotyledons, of which 178 were conserved and 126 non-conserved. They belong to 35 conserved and 113 non-conserved miRNA families. The conserved cotyledon miRNAs had larger family sizes, higher accumulation levels and higher precision rates than non-conserved cotyledon miRNAs. Interestingly, 140 out of the 174 previously identified cotyledon miRNAs were conserved while only 38 out of 130 newly identified cotyledon miRNAs were conserved miRNAs. Thus, the majority of the newly identified miRNAs arose after the soybean speciation. Some of the non-conserved miRNAs are likely to have evolved soybean-specific functions. It is intriguing to understand their functions in soybean.

### Conservation of Soybean Cotyledon miRNAs across Land Plants

**Figure 2:**
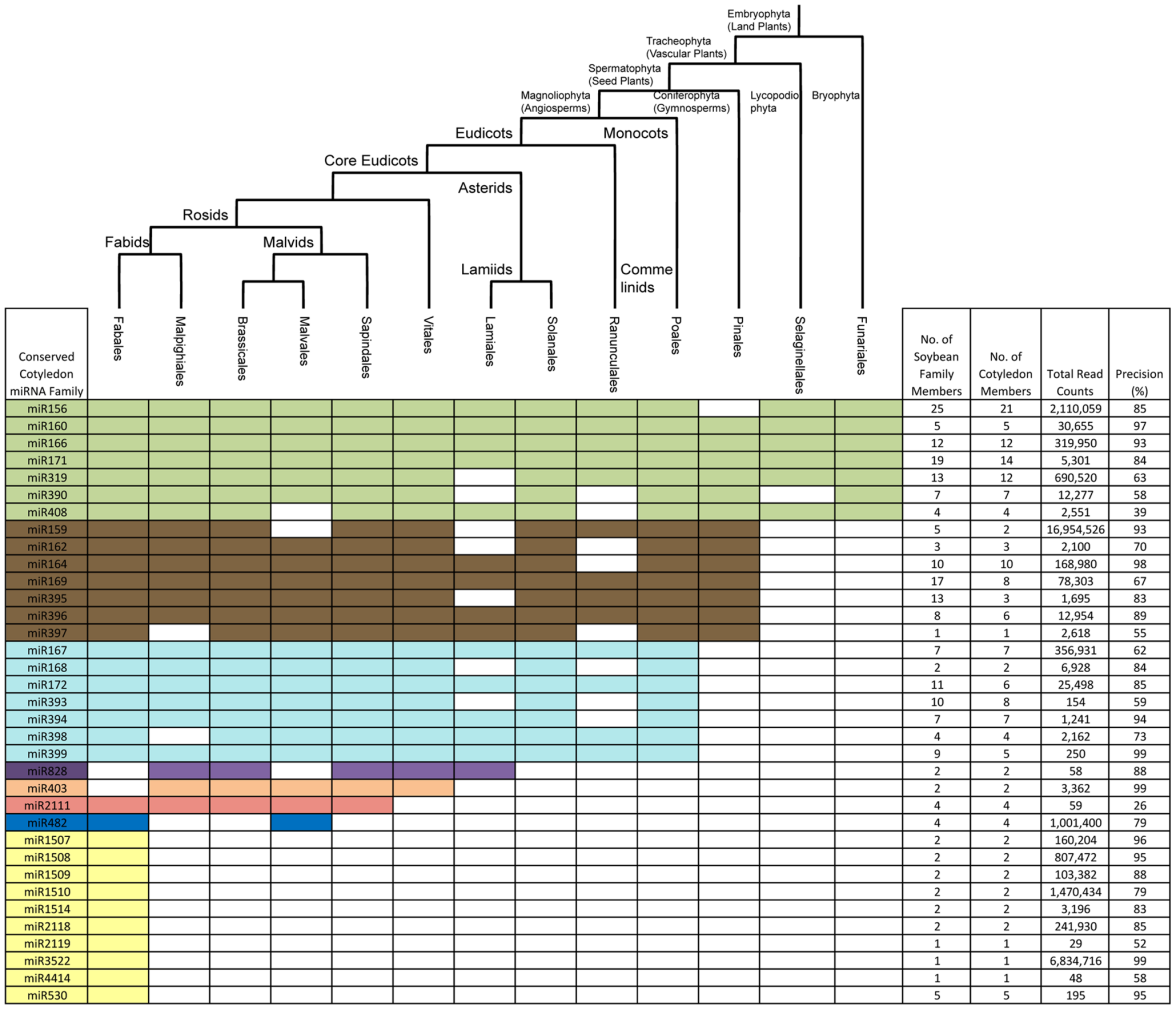
Conserved Soybean Cotyledon miRNAs in Divergent Orders of Land Plants. The conservation of soybean cotyledon miRNA families across plant species belonging to 12 orders is presented. The presence of a miRNA family that is conserved with a soybean cotyledon miRNA in at least one plant species in a given order is shown by differently colored cells. Green cells indicate conservation in land plants, brown cells in seed plants, light blue cells in angiosperms, purple cells in core eudicots, tan cells in vitales, red cells in sapindales, blue cells in rosids and yellow cells in fabales. The evolutionary relationship among the 13 orders is illustrated by the phylogenetic tree placed on top of the miRNA table. The number of family members in the soybean genome and the expressed family members in cotyledons, the count of miRNA reads in cotyledons, and the processing precision are shown for each conserved miRNA family.

A total of 35 conserved miRNA families were expressed in soybean cotyledons. To estimate their conservation across the plant kingdom, we determined their presence in 13 orders of plant species that diverged from the soybean lineage between less than 1 million years ago and more than 1 billion years ago (Figure 2). None of the miRNA families were found in the unicellular green alga *Chlamydomonas reinhardtii*, which was consistent with previous reports (Molnar et al., 2007; Zhao et al., 2007; Cuperus et al 2011). miR156, 160, 166, 171, 319, 390, and 408 represented the most ancient miRNA families. They were present in almost all land plant orders (embryophyta) including funariales, which experienced more than 400 million years of divergent evolution (Figure 2). Seven miRNA families (miR159, miR162, miR164, miR169, miR395, miR396, and miR397) most likely evolved in the common ancestor of spermatophytes, as they were present in both gymnosperm and angiosperm lineages. Seven miRNA families (miR167, miR168, 172, 393, 394, 398 and 399) were angiosperm-specific and present in both eudicots and monocots. miR828, miR403, miR2111, and miR482 were identified in core eudicots, and miR482 is likely restricted to the rosids. Ten miRNA families (miR530, 1507, 1508, 1509, 1510, 1514, 2118, 2119, 3522, and 4414) were only observed in species belonging to the fabales order such as wild soybean, common bean and Medicago, suggesting that these are the youngest conserved miRNA families in soybean. miR403, and miR828 had not been identified in any other species in fabales, but were present in plant species belonging to other eudicot orders. Those miRNAs likely had an ancient origin, but were lost or had not been identified yet in other species in fabales. Considering that miRNAs function as one of key regulatory components in gene regulatory networks, the angiosperm and fabales-specific miRNAs could play important roles in evolving their distinct biological characteristics in cotyledons.

The size of miRNA families in soybean tended to correlate with their evolutionary age (Figure 2). The most ancient miRNA families that evolved in the common ancestors of land plants had a median of 12 miRNA genes per family while the fabales-specific miRNA families only contained a median of 2 miRNA members per family, suggesting that the size of miRNA families expanded over their long evolutionary history. Such an association had been previously observed in Arabidopsis [42], [43]. Although most miRNA genes in each conserved miRNA family were expressed in soybean cotyledons, some of the miRNA genes did not have miRNA reads accumulating in cotyledon tissue. For example, we detected reads for only 2 out of 5 miR159 loci in soybean cotyledons (Table S1 in File S1 and Table S2 in File S1). Although conserved miRNAs had higher accumulation levels than non-conserved miRNAs in soybean cotyledons as shown earlier (Table 1), there was no strong correlation between evolutionary age and accumulation of miRNAs for the conserved miRNA families in cotyledons. Fabales-specific miRNA families had a median accumulation of 131,793 reads per miRNA family while the miRNA families conserved in all orders of embryophyta had a median accumulation of 30,656 reads per miRNA family (Figure 2). miRNA processing has been shown to be more precise in ancient miRNAs than recently arisen miRNA families [43], [44]. We did not find such an obvious correlation between evolutionary age and miRNA processing precision in conserved soybean cotyledon miRNA families. However, as previously reported, more miRNAs from ancient families were 21nt long compared to young, fabales-specific miRNA families [33], [43], [45].

### Characterization of miRNA Loci in the Recently Duplicated Homoeologous Genome Segments

**Figure 3:**
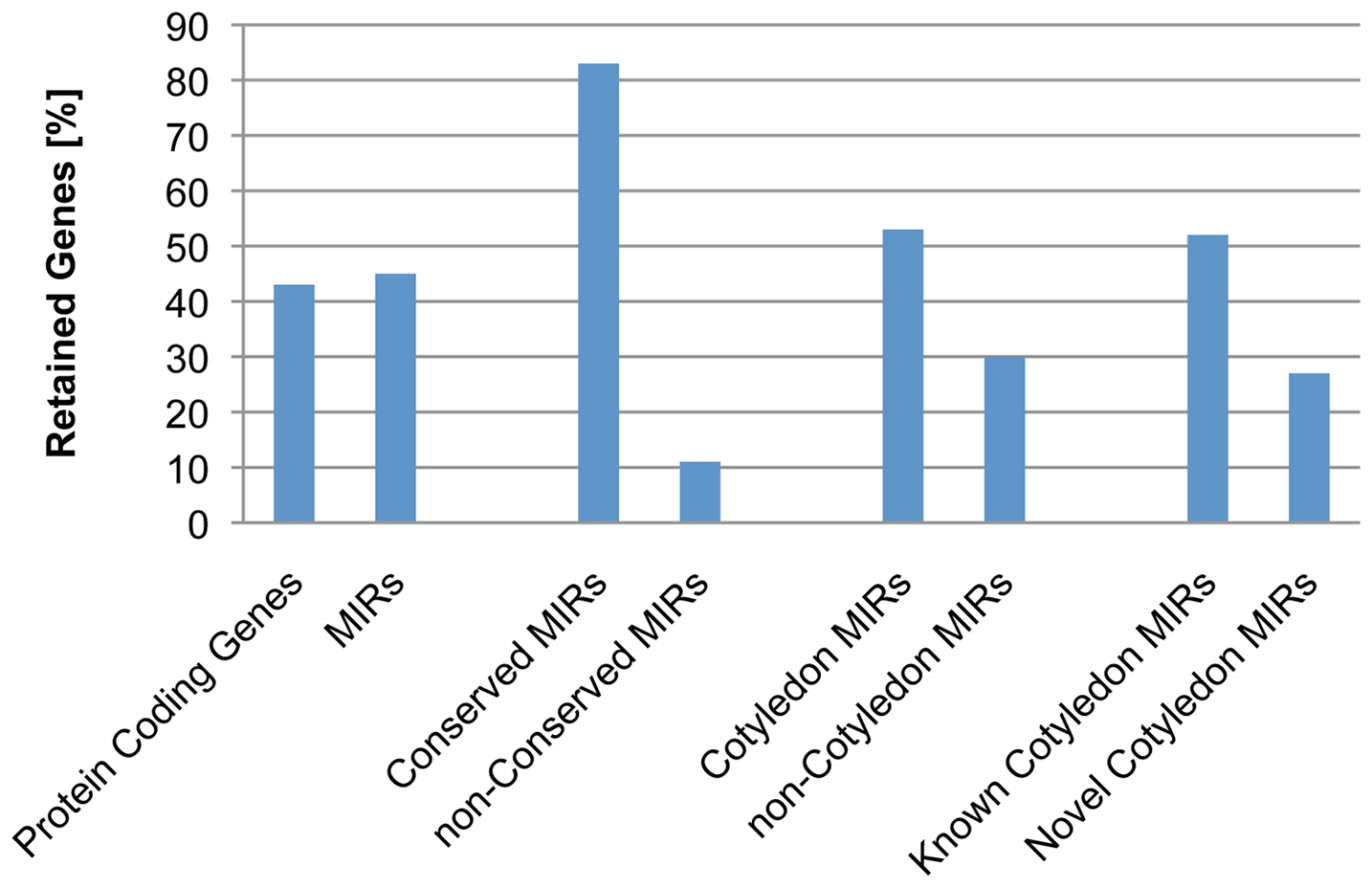
Retention of miRNAs on Recently Duplicated Soybean Genome Segments. The percentage of miRNA genes located on duplicated genome segments that are retained in their paired homologous genome segments from the *Glycine* whole genome duplication is shown on the Y-axis. Each category of miRNAs is indicated on the X-axis.

Soybean is an allotetraploid species. The most recent whole genome duplication occurred about 13 million years ago. One hundred fifty pairs of homoeologous genome segments have been retained from the whole genome duplication event. A total of 419 miRNA loci were located on 172 homoeologous segments belonging to 104 pairs (Table S3 in File S1). Out of the these 419 miRNA loci, 190 miRNA loci (45%) were retained on both duplicated segments while 229 miRNA loci (55%) lost a corresponding miRNA on a homoeologous segment or were newly evolved after the whole genome duplication (Table S4 in File S1). It was observed that 43.4% of protein-coding genes were retained in duplicated segments [27]. Thus, miRNA genes had a rate of loss/gain similar to that of protein-coding genes (Figure 3).

We further determined if conserved miRNAs and non-conserved miRNAs were biasedly retained in duplicated genome segments. One hundred ninety-eight out of the 417 miRNA loci located on duplicated segments were conserved miRNAs. Approximately 83% of the conserved miRNA loci were retained in both paired segments. In contrast, a total of 221 non-conserved miRNA loci were mapped to the duplicated segments, but only 11% of them were retained in both paired segments (Figure 3). Thus, a larger number of non-conserved miRNA loci were either gained or lost after the whole genome duplications. This is consistent with the observation that miRNA loci undergo a frequent birth and death [46]. In addition, 53% of cotyledon miRNAs and 30% of non-cotyledon miRNAs from duplicated segments were retained, which suggests there is a bias in gaining/losing cotyledon miRNA loci in the homoeologous segments. Interestingly, only 27% of novel cotyledon miRNA loci and 52% of known cotyledon miRNA loci from the duplicated segments were retained in both paired segments (Figure 3). Therefore, the newly identified novel cotyledon miRNA loci were preferentially lost or gained after the whole genome duplication. Considering that the non-conserved miRNAs had no orthologs in its closest species such as *Phaseolus vulgaris*, which shared the most recent ancestor with soybean 19 million years ago [47], and majority of those novel miRNAs were non-conserved, it is likely that most of the non-conserved novel miRNAs were gained through a recent birth after the whole genome duplication.

### Recent Birth of the Soy-miR15/49 Family from an Inverted Duplication of a Neutral Invertase Gene

**Figure 4:**
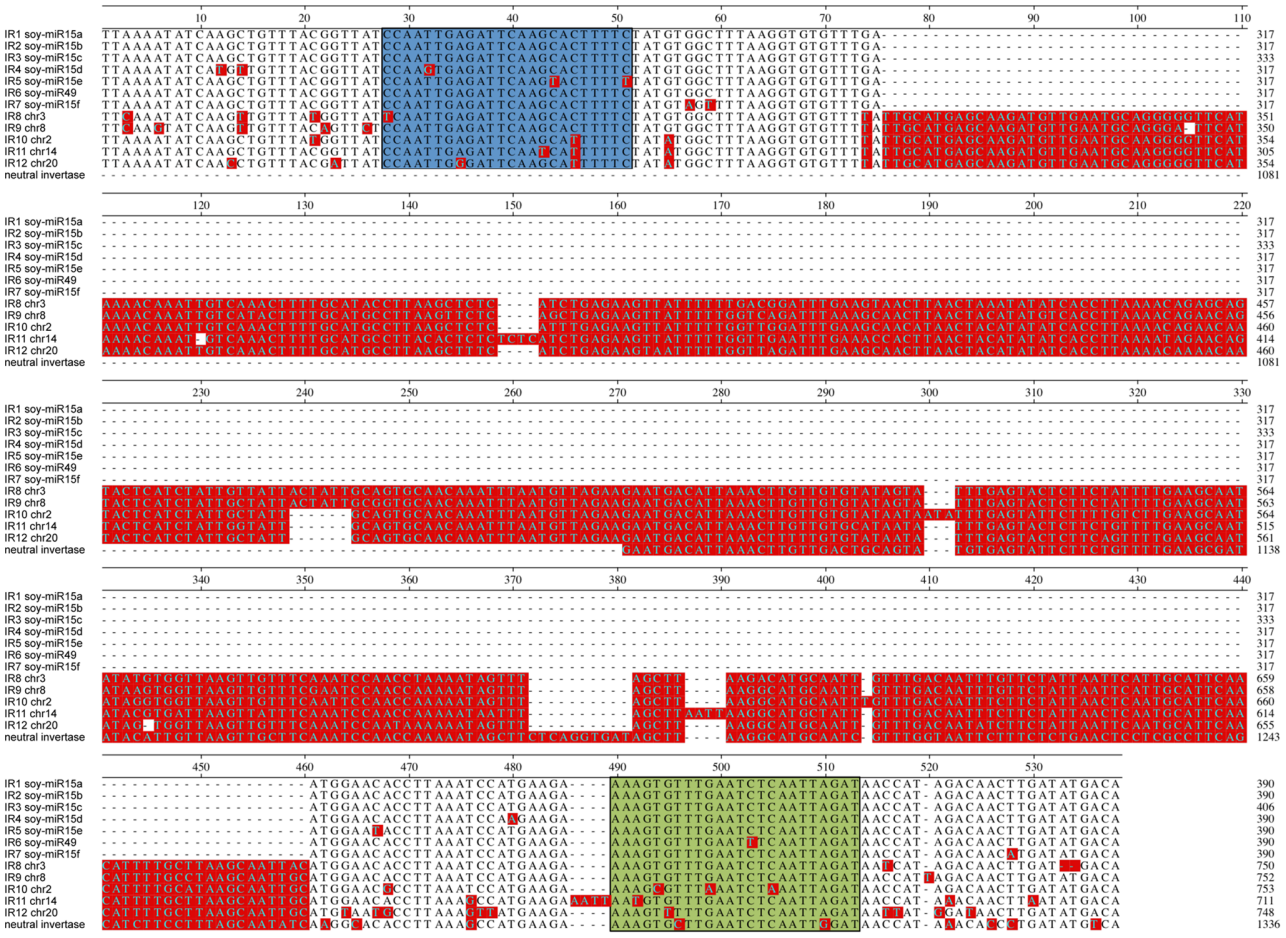
Sequence Alignment of Inverted Repeats and a Neutral Invertase Gene. The central regions of the inverted repeat sequences 1 to 8, their homologous IR sequences from chromosomes 8, 2, 14 and 20 and a fragment of a neutral invertase gene (Glyma12g02690) were aligned. The miRNA and miRNA star sequences are highlighted in green and blue, respectively. Nucleotides that differ from the consensus sequence are shaded in red.

We discovered a novel soybean cotyledon miRNA family, soy-miR15/49. soy-miR15 and soy-miR49 mature sequences differed in one nucleotide. All six soy-miR15 genes and the soy-miR49 gene were located on a 70 kb long region on chromosome 3 (Figure S2). Each of these miRNA genes was situated within a long inverted repeat (IR), and shared strong sequence similarities with each other (Figure 4). The seven inverted repeats (IR1-7) were approximately 647 nucleotides long. In addition, we identified a longer inverted repeat (IR8) and six truncated sequences homologous to IR1-7 that were interspersed with IR1-7 on a 110 kb region of chromosome 3 (Figure S2). The highly repetitive nature of this 110 kb region was further supported by the presence of a 12 kb direct repeat containing a solo LTR and a gene encoding a reticulon protein (Figure S2). Thus, the 110 kb sequence region represents a hot spot of DNA duplication and rearrangement. A total of 657 soy-miR15 reads and 2324 soy-miR49 reads were detected in cotyledons (Table S5 in File S1). All soy-miR15/49 genes had high miRNA processing precision rates, which range from 77% to 92%, further supporting the authenticity of soy-miR15 and 49 (Table S5 in File S1 and Table S6 in File S1).

**Figure 5:**
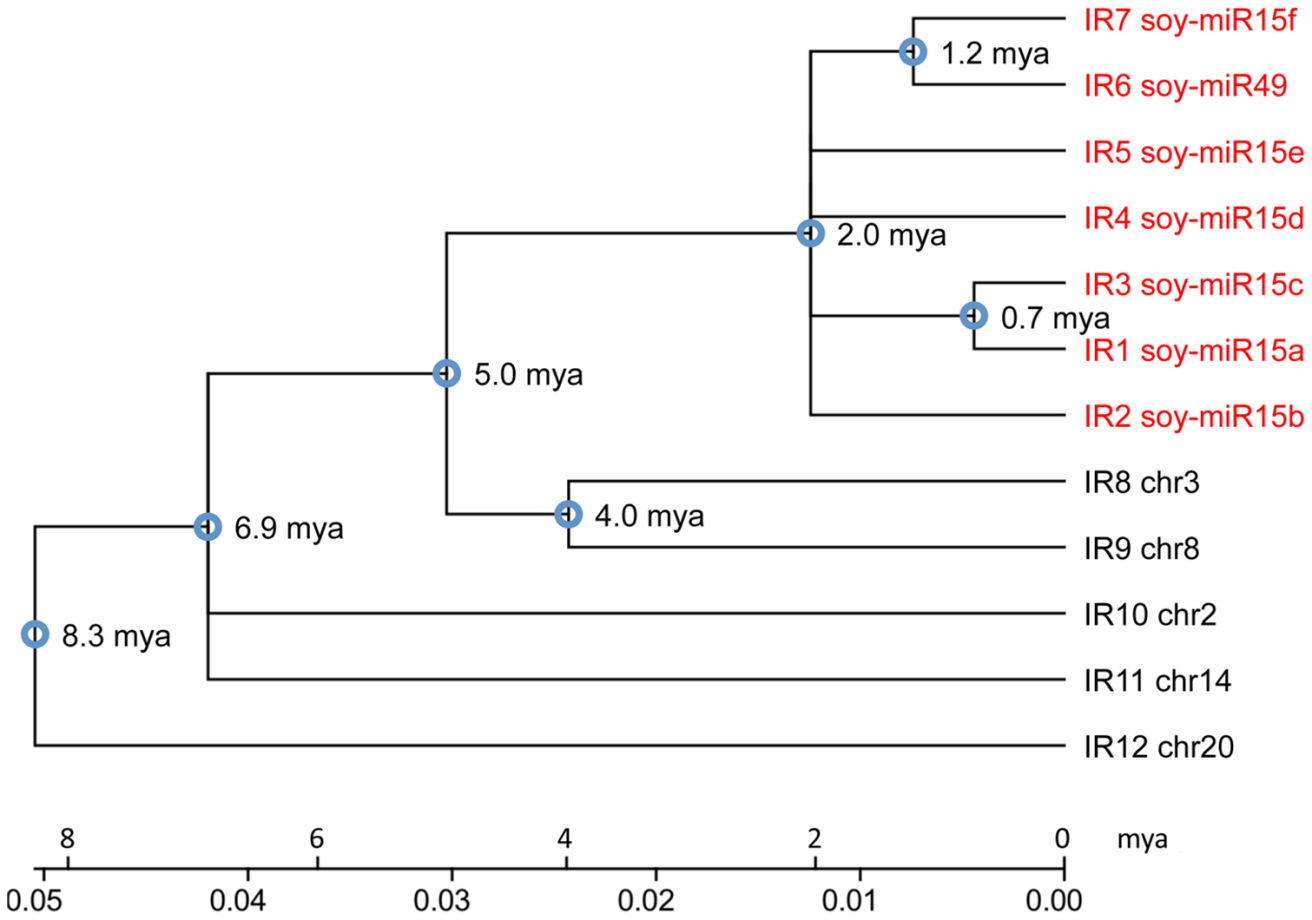
Phylogenetic Relationship of the Inverted Repeat Sequences. The total length of the inverted repeat (IR) sequences 1 to 8 and their homologous IR sequences located on chromosome 2, 8, 14 and 20 were used to construct the phylogenetic tree. IR sequences 1–7 potentially give rise to miRNAs in cotyledons. The time of divergence among the inverted repeats is shown at the tree nodes.

We identified four additional longer inverted repeats (IR9-12) on chromosome 2, 8, 14 and 20 that were nearly identical to IR8 on chromosome 3. All of the longer inverted repeats had strong sequence similarities with IR1-7, but contain a 363 bp insertion sequence at their center (Figure 4). The insertion also formed an inverted repeat sequence, which increased the stem length of their hairpin structures. Our phylogenetic analysis revealed that these IRs arose at least 8.3 million years ago (Figure 5). IR1-7, which lacked the insertion/deletion (indel), clustered in one clade. They probably amplified on chromosome 3 through multiple recombination events over the past two million years. All other IRs, which contained the indel and diverged earlier, likely represent the ancestral IR structure. However, the indel-containing IR8 and IR9 were in the same clade and closely related to the IR1-7. Both clades originated about 5 million years ago. Thus, the indel was likely deleted from the ancestral IR1-7 sequence between 2 to 5 million years ago. Although the inverted repeat (IR1-8) cluster on chromosome 3 was located on one of the duplicated genome segments (ID: 22835160) retained from the *Glycine* whole genome duplication, which occurred 13 million years ago, no homologous IR was observed in the corresponding duplicated genome segment. A gain of the IRs after the whole genome duplication event on just one of the duplicated segments was consistent with the phylogenetic analysis that soy-miR15/49 family arose after the soybean speciation, which occurred 5–10 million years ago [27], [48].

**Figure 6:**
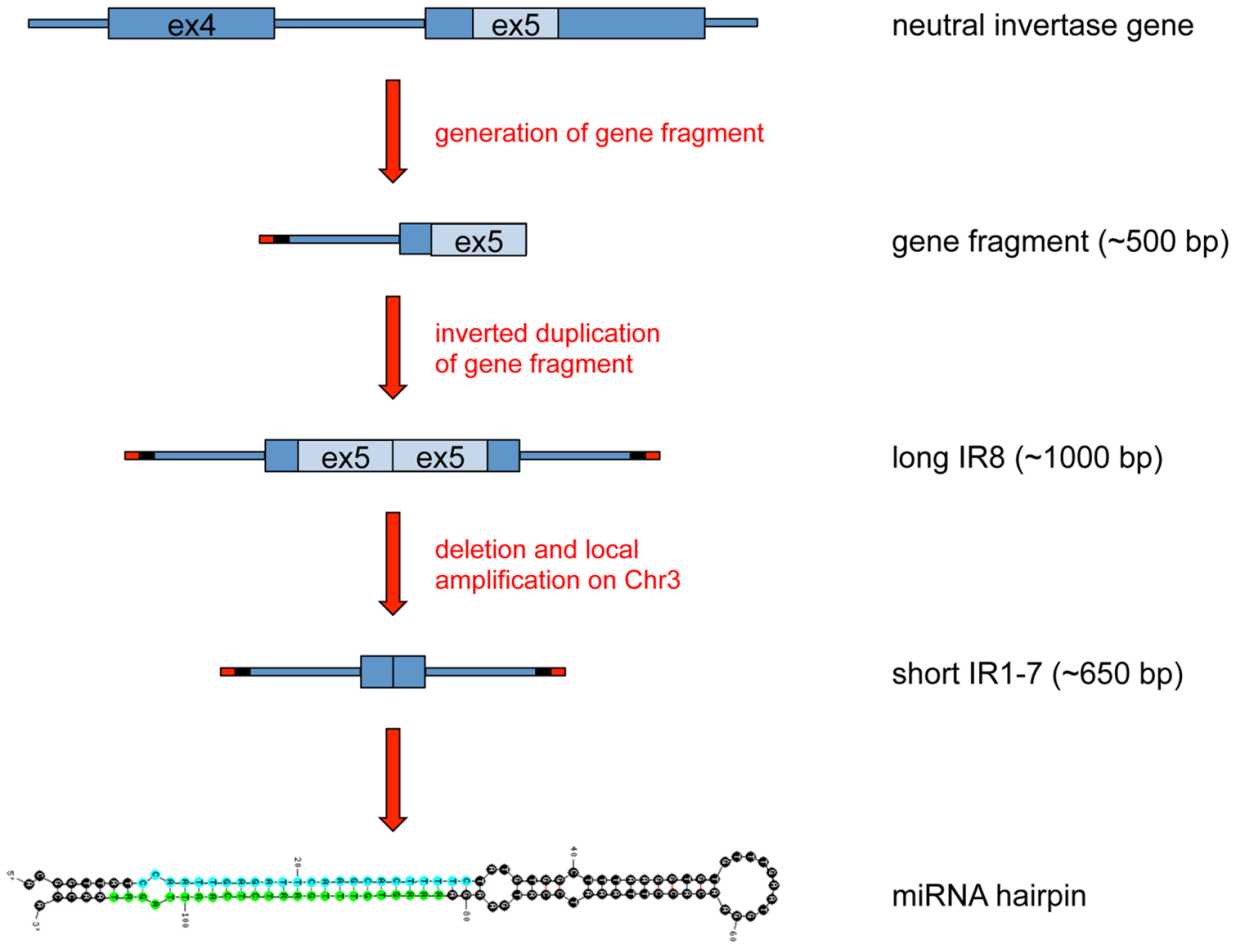
Model Depicting the Evolution of the Soy-miR15/49 Family from an Inverted Duplication of a Neutral Invertase Gene Fragment. A neutral invertase gene fragment containing parts of its intron 4 (blue line) and exon 5 (blue and light blue rectangle) was linked to a short LTR terminal fragment (red line) and a short sequence of unknown origin (black line). This complex DNA arrangement underwent an inverted duplication event, which resulted in a long inverted repeat fragment (IR). Subsequently, this long IR was amplified and transposed to different chromosomes. One copy on chromosome 3 experienced a deletion of a short inverted sequence (light blue rectangles) at its center region, thereby generating a shortened IR structure. This shortened IR went through a series of local amplification events to produce IR1-7, which further evolved to the soy-miR15/49 family. The hairpin structure of *MIR15a* and its miRNA and miRNA* are shown at the bottom.

Interestingly, the inverted repeat units containing the insertion were highly homologous to part of exon 5 (∼240 bp) and intron 4 (∼210 bp) of a gene encoding a neutral invertase (Glyma12g02690). Their terminal sequences contained a 38nt sequence of unknown origin and a 25nt sequence homologous to the same LTR family that was found in the 12 kb direct repeats (Figure 6 and Figure S1). This patchwork of sequences was reminiscent of filler DNA that results from the repair of chromosome breaks through non-homologous end-joining. Thus, we propose that the soy-miR15/49 family most likely evolved recently from the neutral invertase gene (Glyma12g02690) through complex DNA rearrangements and amplifications. The DNA fragment was duplicated and joined in a tail-to-tail fashion to form an inverted repeat structure. This inverted repeat was amplified and spread to chromosomes 20, 14, 2 and 8 and 3 over the past 8.3 million years. Subsequently, the ancestral inverted repeat of IR1-7 lost the 360 bp indel sequence between 2 to 5 million years ago and was later amplified through a series of local DNA duplications until the IR cluster reached its current shape. We did not detect miRNA reads aligning to IR10-12. It is likely that the miR15/49 family arose after the split of the IR1-7 clade from the IR8-9 clade. Deletion of the 360 bp long indel significantly decreased the distance between the miR15/miR15* duplex and the loop of the hairpin structure, and might promote the birth of the soy-miR15/49 family. soy-miR15/49 represented a young and recently evolved miRNA family, and still maintained a strong sequence similarity to the neutral invertase gene. The length of the duplicated soy-miR15 gene sequences extends beyond their miRNA fold-back structure, which has been reported for many Arabidopsis miRNA genes as well [19]. It has also been proposed that *MIR* genes can evolve from inverted duplicates of protein-coding genes or gene segments [19], [21], [46], [49]–[52]. Initially, these perfect inverted repeats would produce hairpin transcripts that are processed by DCL4 and DCL3 to yield 24nt siRNAs. Over time, drift mutations would disrupt the perfect hairpin structures that would cause the transition from DCL4 to DCL1 cleavage resulting in the production of 21nt miRNAs. Further accumulation of mutations over time in regions surrounding the miRNA/miRNA* sequence could generate miRNA genes that have lost similarities with the parental donor sequence and therefore may appear unrelated to their locus of origin. Our in-depth analysis of the soy-miR15/49 family not only provided strong evidence for the hypothesis that miRNAs can evolve from inverted duplications of protein-coding gene fragments, but also illustrated the evolutionary pathway that gave birth to the miR15/49 family.

### Complex Networks Underlying Interaction of Cotyledon MicroRNAs and Their Target Transcripts

Plant miRNAs mainly interact with protein-coding transcripts containing perfect or near-perfect complement sequences, and further cause the degradation of their target transcripts in plant cells. Such miRNA-transcript interactions form a core regulatory network in gene regulatory programs. A TargetFinder algorithm was used to query all known soybean protein-coding transcripts with 148 cotyledon miRNA families to predict genes that are regulated by cotyledon miRNAs [46]. Out of 148 cotyledon miRNA families, 130 miRNA families were predicted to target at least one gene. Approximately 16% of the miRNA-transcript interactions occurred at 5′ or 3′ UTRs (Table S7 in File S1). The 130 cotyledon miRNA families were predicted to target 1910 protein-coding transcripts, which form a complex network system containing 2021 interactions (Figure S3). Cotyledon miRNA families had an average of 16 targets with a range of 1 to 124 targets per miRNA family (Table S8 in File S1). The newly identified and non-conserved soy-miR38 family had the highest number of predicted targets (Table S9 in File S1).

**Figure 7:**
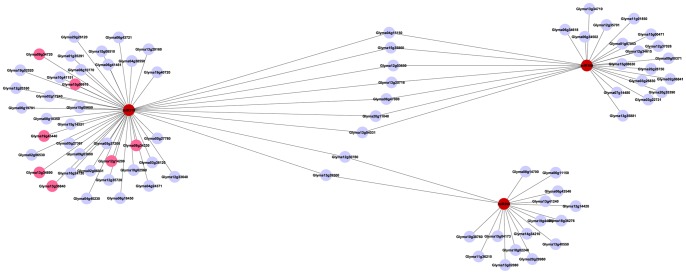
Interactions of miR395, 319 and 159 with Their Target Genes. Interactions of miR395, 319 and 159 (red circles) with their target genes (light blue circles and pink circles) are shown. Two targets are commonly regulated by miR319 and miR395 while seven targets are commonly regulated by miR319 and miR159.

The majority of targets specifically interacted with a single miRNA family. However, 81 genes were targeted by multiple miRNA families, which formed 192 distinct gene-miRNA family interactions. Thirty-seven genes were targeted by 42 cotyledon miRNA families at more than two distinct sites (Table S10 in File S1). Shared target genes connected miRNA modules together to form compound modules (Figure 7 and Figure S3), which increased the complexity of the miRNA-target network system. For example, miR319 shared two targets with miR395 and seven targets with miR159 (Figure 7). All three miRNA families are ancient (Figure 2). miR319 and miR159 shared significant sequence similarity and had been shown to evolve from a common ancestral miRNA gene [53]. Their shared targets were a beta-galactosidase gene, five MYB33 and one MYB65 genes (Table S11 in File S1). It is likely that these interactions predated the divergence of miR159 and miR319 and were conserved through their long evolutionary history. However, thirty-eight genes with a variety of functions were specifically targeted by miR319 while 18 genes were only targeted by miR159. miR159-specific or miR319-specific interactions contributed to their distinct biological functions in soybean cotyledons and are possible outcomes from neo-functionalization or sub-functionalization after the gene duplication event. It has been shown in Arabidopsis that the interaction of miR159 and its target MYB genes is involved in the regulation of vegetative growth, flowering time, seed shape and germination [54]–[56]. In contrast to miR159, miR319 regulates embryonic patterning, jasmonate synthesis, leaf morphogenesis and senescence in Arabidopsis and tomatoes [57]–[59]. Expression patterns of miR159 and miR319 are significantly different in Arabidopsis. Accumulation of miR159 is abundant and widespread over the whole plants, while accumulation of miR319 is mainly confined to specific tissues and developmental stages [53], [57]. Their diverse temporal and spatial-specific expression also contributes to their functional specialization in plant growth and development.

### Networks Underlying Interaction of Cotyledon MicroRNAs and Biological Pathways

We predicted biological pathways and functional groups that are regulated by cotyledon miRNAs. In the analysis, soybean genes targeted by cotyledon miRNAs were assigned into hierarchical functional groups based on their molecular function in metabolic and signaling pathways [60]. A Fisher's Exact Test was used to determine the significance of over- and under-represented miRNA target genes in those functional groups. Cotyledon miRNAs preferentially targeted genes involved in RNA metabolism and the regulation of transcription (Table 3). A large number of transcription factor gene families such as CCAAT box binding factor, TCP, and ARF transcription factor families were preferentially targeted by cotyledon miRNAs. In addition, we also observed that cotyledon miRNAs preferentially targeted a number of other functional groups such as lipid metabolism and major CHO metabolism.

**Table 3:** Over-representation of Cotyledon miRNA Targeted Genes in Biological Pathways.

Functional Groups	p-value	odds ratio
RNA	2.04E-02	1.28
RNA.regulation_of_transcription	2.14E-04	1.57
RNA.regulation_of_transcription.General_Transcription	3.00E-04	7.91
RNA.regulation_of_transcription.ARF_Auxin_Response_Factor_family	3.02E-08	10.31
RNA.regulation_of_transcription.CCAAT_box_binding_factor_family_HAP2	2.57E-05	21.94
RNA.regulation_of_transcription.HBHomeobox_transcription_factor_family	1.02E-03	3.58
RNA.regulation_of_transcription.TCP_transcription_factor_family	8.55E-03	8.75
hormone_metabolism.auxin.signal_transduction	6.64E-03	6.18
signalling.receptor_kinases.misc	2.32E-02	2.63
protein.degradation.ubiquitin.E2	2.11E-02	2.98
amino_acid_metabolism.degradation.glutamate_family.proline	1.26E-02	17.46
fermentation.ADH	1.34E-02	7.15
lipid_metabolism	2.38E-02	1.68
lipid_metabolism.FA_synthesis_and_FA_elongation	3.65E-02	2.22
major_CHO_metabolism.degradation.sucrose.invertases	2.31E-02	5.62
major_CHO_metabolism.degradation.sucrose.invertases.neutral	3.55E-03	13.11
minor_CHO_metabolism.galactose.galactokinases	2.52E-02	10.48
misc.oxidases___copper_flavone_etc	1.08E-02	2.64
misc.plastocyanin_like	2.34E-02	4.04
protein.synthesis.misc_ribososomal_protein.BRIX	3.28E-02	8.73
protein.targeting.secretory_pathway.golgi	2.31E-02	5.62
S_assimilation.APS	1.26E-02	17.46
secondary_metabolism.simple_phenols	1.36E-11	20.42
transport.sulphate	2.69E-02	5.25

Representation analysis of genes targeted by individual cotyledon miRNA families in functional groups was conducted to predict biological pathways a given family preferentially regulated. A total of 101 miRNA families preferentially targeted at least one biological pathway and combine to form complex miRNA-pathway networks (Table S12 in File S1 and Figure S4). We observed nine cotyledon miRNA families, each preferentially regulating one specific biological pathway. The rest of the cotyledon miRNA families preferentially targeted two or more pathways. Additionally, several biological pathways were preferentially targeted by multiple miRNA families (Table S12 in File S1). The one-to-one, one-to-many, and many-to-one miRNA pathway interactions led to a complex regulatory network system composed of interaction modules with variable size in soybean cotyledons (Figure S4).

## Conclusions

Soybean cotyledons serve as specialized sink tissues for the synthesis and storage of seed reserves. They provide nutrients and energy through reserve mobilization for seed germination and seedling growth. We generated and analyzed one of the largest collections of small RNAs with a total of 292 million raw sequencing reads. Soybean cotyledons accumulated at least 41 million unique small RNA species, and therefore had an extremely complex small RNA population. A total of 130 novel miRNAs and 72 novel miRNA families were identified, which significantly increased the collection of currently known soybean miRNAs. We showed that 304 miRNA genes were expressed in soybean cotyledons. Examining the presence of conserved cotyledon miRNAs in 13 divergent orders of land plant species showed that the cotyledon miRNAs arose at various stages over the course of land plant evolution. The size of conserved miRNA families tend to correlate with their evolutionary age in soybean. Ancient miRNAs typically had a larger family size than young miRNAs. Although conserved cotyledon miRNAs had the tendency to be accumulated at higher read levels than non-conserved cotyledon miRNAs, there was no obvious correlation between accumulation level and age of conserved miRNAs.

It has been proposed that miRNA genes could evolve from inverted duplications of their target genes. However, evolutionary mechanisms for such a miRNA origin largely remain unknown. We conducted a detailed structural and phylogenetic analysis of the soy-miR15/49 family and illustrated that the soy-miR15/49 family probably evolved from an inverted duplication of a protein-coding gene fragment through transposition of the inverted repeats to different chromosomes, local tandem amplifications, fragment deletion and nucleotide mutations. Bioinformatic and statistical analysis revealed that cotyledon miRNAs target genes encoding proteins with diverse biological functions. The genes coding for proteins involved in RNA metabolism, regulation of transcription and signaling pathways are preferentially targeted by miRNAs. Thus, miRNAs are likely to regulate a variety of biological pathways in cotyledons and play central roles in their underlying gene regulatory networks. One-to-one, one-to-many, many-to-one and many-to-many interactions of miRNA-target gene/pathways were identified, which could further form a complex regulatory network system composed of interaction modules of variable size in soybean cotyledons. The topology of the miRNA-gene and miRNA-pathway networks provides a comprehensive view into their complex and intricate interactions at a systems level and lays a foundation to design a variety of wet-bench experiments for functional validation.

## Materials and Methods

### Plant Materials and Library Construction

Soybean (*Glycine max* (L.) Merrill, cv. Jack) plants were grown in growth rooms with supplemental lighting. Cotyledons were isolated from seeds at six developmental stages (S2, 3, 4, 6, and 8) with three replications. Total RNA was isolated as described by Chen and An [61] with minor modification. 17–30nt long RNA was purified by polyacrylamide gel electrophoresis to construct cDNA libraries. A total of 18 cDNA libraries were generated and sequenced by Expression Analysis, Inc. (Durham, NC) using the SBS (sequencing by synthesis) technology.

### Initial processing of sequencing libraries

Raw sequence reads with no 3′ sequencing adaptor, of low quality, or shorter than 17nt were removed. The adaptor trimming was done by an in-house method that recursively searches for the longest substring of the adaptor appearing within a sequence read. If a raw sequence read did not have a substring of the adaptor longer than 6nt, it was considered as having no adaptor. The adaptor-trimmed sequences with no ambiguous reads, referred to as qualified reads, were then mapped, allowing for 0 mismatches, to the soybean genome using Bowtie [62].

### Identification of novel miRNAs

Novel miRNAs were identified by following the previously described method with minor revisions [63] [64]. Briefly, qualified reads in all libraries that mapped to known soybean miRNAs in miRBase release 20, rRNA, and tRNA were excluded from the analysis. We then mapped the remaining reads to the soybean genome using Bowtie [27], [62], [65] and merged neighboring loci if they shared overlapping reads. Subsequently, we examined the folding structures of the (merged) loci. Since the average length of a miRNA precursor in plants is often ∼200nt, we took 250nt as the length of putative pre-miRNAs in our analysis. At each genomic locus to be analyzed, a series of DNA sequence segments covering the sequence reads were extracted for secondary structure analysis. The starting sequence segment extended 220nt upstream of the sequence reads, and subsequent segments were extracted by a sliding window of 250nt, with an increment of 10nt, until the window reached 220nt downstream of the sequence reads. Each of these individual segments was folded by the RNA-fold program [66]. A segment lacking a stem of at least 18nt or a segment lacking sequencing reads that map to any of its stems was excluded. A representative segment was chosen from those that have the same or a similar folding structure. The top five folding structures with a free energy no greater than -18 kCal/mol were further visually inspected. We retained those segments that formed a typical hairpin fold-back structure with up to 5 mismatches on one stem region. Finally, a program, which encoded the four miRNA-annotation rules described below, was adopted to scan each segment. Candidate miRNAs were chosen based on the criteria: 1) occurrence of miRNA reads on the arms of predicted hairpin structures; 2) presence of no less than 10 miRNA reads of the highest frequency on predicted hairpins; 3) presence of possible miRNA* sequencing reads unless specifically stated (see below) 4) presence of 2–3nt 3′ overhangs on miRNA/miRNA* duplexes [38]. After miRNA were derived computationally, they were subjected to a manual review. All small RNA reads were aligned to their corresponding hairpin sequence and visually inspected. The read with the highest read count, cumulatively from all libraries, was preferentially selected as the mature miRNA sequence. The count of a miRNA or a miRNA* was calculated solely based on the reads aligning to its mature miRNA sequence or miRNA* sequence. The iso-miRNA reads were not counted. The secondary structure for each hairpin was also visualized to verify the star sequence. Any hairpins with more than 5 mismatches in their miRNA/miRNA* duplexes were removed from the set. Neither the mature nor the star sequence could be located in the loop. The hairpin loci were compared to one another to see which were overlapping. The hairpin with the highest expression and/or greatest precision was selected in the cases where overlaps were noted and the others were removed from the set. A low stringency cut off of 10% processing precision was applied to all miRNA. miRNAs with no star sequences were removed if they showed less than 45% precision. Processing precision was calculated as ((mature read count) + (star read count)/total count of reads aligned to hairpin) * 100.

### Target Gene Prediction and Validation

The TargetFinder program release 1.6 (http://jcclab.science.oregonstate.edu/node/view/56334) was used to query all known and novel soybean miRNA sequences against all annotated soybean transcript sequences from Gmax_109_transcriptome.fa in Phytozome v9.1 at the cutoff alignment score of 4. Soybean genes were binned in MapMan functional groups by converting the respective gene ID to Affymetrix probeset IDs. The total number of binned target genes and soybean genes in each of the functional groups were input to a Fisher's exact test using an R script to determine the p-values for over or under-representation of target genes. Custom Perl scripts were used to parse the odds ratios and p-values.

### Mapping and displaying miRNA loci and targeted genes

pre-miRNA coordinates for all known and novel soybean miRNA loci were determined by BLAST searches against the genomic soybean reference sequence in Phytozome v9.1. The pre-miRNA genomic sequence coordinates were written in a gff file format and then loaded to and viewed in the SoyBase Genome Browser (http://www.soybase.org/gb2/gbrowse/gmax1.01/). A custom Perl script was used to identify pre-miRNA sequences on regions of recent segmental duplication defined in ftp://ftp.jgi-psf.org/pub/JGI_data/phytozome/v6.0/Gmax/related_data/recentDuplSegments.gff3.

### Phylogenetic analysis

Sequences were aligned using ClustalW in MEGA5 [67], and the resulting alignment was manually adjusted. The evolutionary history was inferred using the Neighbor-Joining method [68]. The optimal tree with the sum of branch length  = 0.30150857 is shown. The evolutionary distances were computed using the Kimura 2-parameter method [69] and are in the units of the number of base substitutions per site. Evolutionary analyses were conducted in MEGA5 [67]). The divergence time points of the inverted repeats were calculated in MEGA5 [67] using the substitution rate R of 6.1×10^−9^ mutations per site per year [70].

## Supporting Information

Figure S1 **Average Accumulation Distribution of Small RNAs at Each Size in Soybean Cotyledons.** The average count of reads per unique small RNA at each given size in cotyledon tissues is indicated on the Y-axis. The X-axis indicates the size of small RNAs.
(PDF)

Figure S2 **Soy-miR15/49 Gene Cluster Region.** A 110 kb region on chromosome 3 contains multiple miRNA genes of the soy-miR15/49 family. Each of their miRNA fold-back structures are embedded in inverted repeat sequences (IR1-7) drawn as blue diamonds to represent their inverted structures. The inverted repeat sequence containing an indel in its middle region is illustrated as an orange diamond (IR8). The phylogenetic tree presented on the top indicates the evolutionarily relationship among those IRs. Truncated IRs homologous to the full-length IRs are shown as green triangles. Four genes (gray and purple rectangles) are located in this repetitive region. The reticulon protein genes are embedded in the 12 kb duplicated regions (light blue rectangle). The direct repeat region also contains a solo LTR (black rectangle).
(PDF)

Figure S3 **Global Topology of miRNA-target Networks.** The conserved and non-conserved miRNAs are represented by red and blue circles, respectively. Target genes that were categorized into functional bins are shown. Targets encoding proteins related to RNA metabolism (pink circles), Ubiquitin based protein degradation (green circles), receptor kinase families (orange circles), secondary metabolism (turquoise circles), lipid metabolism (yellow circles) are distinguished from all other targets (light blue circles). Thick black edges connecting nodes indicate network overrepresented by cotyledon miRNA targets in preferentially regulated biological pathways.
(PDF)

Figure S4 **Global Topology of miRNA-Biological Pathway Networks.** The conserved and non-conserved miRNAs were indicated by red and blue circles, respectively. Their targeted biological pathways are shown by pink circles representing biological pathways related to RNA metabolism, green circles standing for Ubiquitin based protein degradation pathways, orange circles denoting receptor kinase families and light blue circles representing all remaining pathways.
(PDF)

File S1 **This file contains: Table S1. Known miRNAs Expressed in Soybean Cotyledon. Table S2. Novel Cotyledon miRNAs. Table S3. Summary of miRNAs in Duplicated Genome Segments. Table S4. Summary of miRNA loci in Recently Duplicated Genome Segments. Table S5. Read Count and Precision Rate for Soy-mir15/49. Table S6. Reads Aligned to the Hairpin Sequences of Each Soy-miR15/49. Table S7. Cotyledon Soybean miRNA Families and Their Putative Target Genes. Table S8. Summary of Cotyledon miRNA Family Targeted Genes. Table S9. Summary of Soy-Mir38 Targeted Genes. Table S10. Genes Targeted by Multiple Cotyledon miRNA at Distinct Locations. Table S11. Genes Targeted by Mirna319, 395 and 159 Families. Table S12. Over- or Under-Representation of Genes Targeted by Cotyledon miRNA Family in Each Functional Bin.**
(XLSX)

## References

[1] Lee Y, Kim M, Han J, Yeom KH, Lee S, et al (2004) MicroRNA genes are transcribed by RNA polymerase II. EMBO J 23: 4051–4060. 15372072 10.1038/sj.emboj.7600385 PMC524334

[2] Zhou X, Ruan J, Wang G, Zhang W (2007) Characterization and identification of microRNA core promoters in four model species. PLoS Comput Biol 3: e37. 17352530 10.1371/journal.pcbi.0030037 PMC1817659

[3] Carthew RW, Sontheimer EJ (2009) Origins and Mechanisms of miRNAs and siRNAs. Cell 136: 642–655. 19239886 10.1016/j.cell.2009.01.035 PMC2675692

[4] Bartel DP (2004) MicroRNAs: genomics, biogenesis, mechanism, and function. Cell 116: 281–297. 14744438 10.1016/s0092-8674(04)00045-5

[5] Kim VN (2005) MicroRNA biogenesis: coordinated cropping and dicing. Nat Rev Mol Cell Biol 6: 376–385. 15852042 10.1038/nrm1644

[6] Vaucheret H (2008) Plant ARGONAUTES. Trends in Plant Science 13: 350–358. 18508405 10.1016/j.tplants.2008.04.007

[7] Okamura K, Liu N, Eric C, Lai EC (2009) Distinct Mechanisms for MicroRNA Strand Selection by Drosophila Argonautes. Mol Cell 36: 431–444. 19917251 10.1016/j.molcel.2009.09.027 PMC2785079

[8] Rhoades MW, Reinhart BJ, Lim LP, Burge CB, Bartel B, et al (2002) Prediction of plant microRNA targets. Cell 110: 513–520. 12202040 10.1016/s0092-8674(02)00863-2

[9] Carrington JC, Ambros V (2003) Role of microRNAs in plant and animal development. Science 301: 336–338. 12869753 10.1126/science.1085242

[10] Bartel DP (2004) MicroRNAs: genomics, biogenesis, mechanism, and function. Cell 116: 281–297. 14744438 10.1016/s0092-8674(04)00045-5

[11] Dugas DV, Bartel B (2008) Sucrose induction of Arabidopsis miR398 represses two Cu/Zn superoxide dismutases. Plant Molecular Biology 67: 403–417. 18392778 10.1007/s11103-008-9329-1

[12] Brodersen P, Sakvarelidze-Achard L, Bruun-Rasmussen M, Dunoyer P, Yamamoto YY, et al (2008) Widespread translational inhibition by plant miRNAs and siRNAs. Science 320: 1185–1190. 18483398 10.1126/science.1159151

[13] Zhai J, Jeong DH, De Paoli E, Park S, Rosen BD, et al (2011) MicroRNAs as master regulators of the plant NB-LRR defense gene family via the production of phased, trans-acting siRNAs. Genes & Development 25: 2540–2553. 22156213 10.1101/gad.177527.111 PMC3243063

[14] Xia R, Zhu H, An YQ, Beers EP, Liu Z (2012) Apple miRNAs and tasiRNAs with novel regulatory networks. Genome biology 13: R47. 22704043 10.1186/gb-2012-13-6-r47 PMC3446319

[15] Palatnik JF, Allen E, Wu X, Schommer C, Schwab R, et al (2003) Control of leaf morphogenesis by microRNAs. Nature 425: 257–263. 12931144 10.1038/nature01958

[16] Aukerman MJ, Sakai H (2003) Regulation of flowering time and floral organ identity by a MicroRNA and its APETALA2-like target genes. Plant Cell 15: 2730–2741. 14555699 10.1105/tpc.016238 PMC280575

[17] Park W, Li J, Song R, Messing J, Chen X (2002) CARPEL FACTORY, a Dicer homolog, and HEN1, a novel protein, act in microRNA metabolism in Arabidopsis thaliana. Curr Biol 12: 1484–1495. 12225663 10.1016/s0960-9822(02)01017-5 PMC5137372

[18] Sunkar R, Zhu JK (2004) Novel and stress-regulated microRNAs and other small RNAs from Arabidopsis. Plant Cell 16: 2001–2019. 15258262 10.1105/tpc.104.022830 PMC519194

[19] Fahlgren N, Jogdeo S, Kasschau KD, Sullivan CM, Chapman EJ, et al (2010) MicroRNA gene evolution in Arabidopsis lyrata and Arabidopsis thaliana. Plant Cell 22: 1074–1089. 20407027 10.1105/tpc.110.073999 PMC2879733

[20] Ma Z, Coruh C, Axtell MJ (2010) Arabidopsis lyrata small RNAs: transient MIRNA and small interfering RNA loci within the Arabidopsis genus. Plant Cell 22: 1090–1103. 20407023 10.1105/tpc.110.073882 PMC2879747

[21] Rajagopalan R, Vaucheret H, Trejo J, Bartel DP (2006) A diverse and evolutionarily fluid set of microRNAs in Arabidopsis thaliana. Genes Dev 20: 3407–3425. 17182867 10.1101/gad.1476406 PMC1698448

[22] Xie Z, Allen E, Fahlgren N, Calamar A, Givan SA, et al (2005) Expression of Arabidopsis MIRNA genes. Plant Physiol 138: 2145–2154. 16040653 10.1104/pp.105.062943 PMC1183402

[23] Chen X (2004) A microRNA as a translational repressor of APETALA2 in Arabidopsis flower development. Science 303: 2022–2025. 12893888 10.1126/science.1088060 PMC5127708

[24] Li H, Deng Y, Wu T, Subramanian S, Yu O (2010) Misexpression of miR482, miR1512, and miR1515 Increases Soybean Nodulation. Plant Physiol 153: 1759–1770. 20508137 10.1104/pp.110.156950 PMC2923892

[25] Jones-Rhoades MW, Bartel DP, Bartel B (2006) MicroRNAs and Their Regulatory Roles in Plants Annual Review of Plant Biology. 57: 19–53. 10.1146/annurev.arplant.57.032905.105218 16669754

[26] Shamimuzzaman M, Vodkin L (2012) Identification of soybean seed developmental stage-specific and tissue-specific miRNA targets by degradome sequencing. BMC Genomics 13: 310. 22799740 10.1186/1471-2164-13-310 PMC3410764

[27] Schmutz J, Cannon SB, Schlueter J, Ma J, Mitros T, et al (2010) Genome sequence of the palaeopolyploid soybean. Nature 463: 178–183. 20075913 10.1038/nature08670

[28] Zhang B, Pan X, Stellwag E (2008) Identification of soybean microRNAs and their targets. Planta 229: 161–182. 18815805 10.1007/s00425-008-0818-x

[29] Subramanian S, Fu Y, Sunkar R, Barbazuk WB, Zhu JK, et al (2008) Novel and nodulation-regulated microRNAs in soybean roots. BMC Genomics 9: 160. 18402695 10.1186/1471-2164-9-160 PMC2335117

[30] Joshi T, Yan Z, Libault M, Jeong D-H, Park S, et al (2010) Prediction of novel miRNAs and associated target genes in Glycine max. BMC Bioinformatics 11: S14. 10.1186/1471-2105-11-S1-S14 PMC3009485 20122185

[31] Song Q-X, Liu Y-F, Hu X-Y, Zhang W-K, Ma B, et al (2011) Identification of miRNAs and their target genes in developing soybean seeds by deep sequencing. BMC plant biology 11: 5. 21219599 10.1186/1471-2229-11-5 PMC3023735

[32] Kulcheski F, de Oliveira L, Molina L, Almerao M, Rodrigues F, et al (2011) Identification of novel soybean microRNAs involved in abiotic and biotic stresses. BMC Genomics 12: 307. 21663675 10.1186/1471-2164-12-307 PMC3141666

[33] Turner M, Yu O, Subramanian S (2012) Genome organization and characteristics of soybean microRNAs. BMC Genomics 13: 169. 22559273 10.1186/1471-2164-13-169 PMC3481472

[34] Kozomara A, Griffiths-Jones S (2011) miRBase: integrating microRNA annotation and deep-sequencing data. Nucleic Acids Research 39: D152–D157. 21037258 10.1093/nar/gkq1027 PMC3013655

[35] Axtell MJ (2013) Classification and Comparison of Small RNAs from Plants. Annual Review of Plant Biology 64: 137–159. 10.1146/annurev-arplant-050312-120043 23330790

[36] Lelandais-Briere C, Naya L, Sallet E, Calenge F, Frugier F, et al (2009) Genome-Wide Medicago truncatula Small RNA Analysis Revealed Novel MicroRNAs and Isoforms Differentially Regulated in Roots and Nodules. Plant Cell 21: 2780–2796. 19767456 10.1105/tpc.109.068130 PMC2768930

[37] Okamura K, Phillips MD, Tyler DM, Duan H, Chou YT, et al (2008) The regulatory activity of microRNA* species has substantial influence on microRNA and 3′ UTR evolution. Nature structural & molecular biology 15: 354–363. 10.1038/nsmb.1409 PMC2698667 18376413

[38] Meyers BC, Axtell MJ, Bartel B, Bartel DP, Baulcombe D, et al (2008) Criteria for Annotation of Plant MicroRNAs. Plant Cell 20: 3186–3190. 19074682 10.1105/tpc.108.064311 PMC2630443

[39] Mi S, Cai T, Hu Y, Chen Y, Hodges E, et al (2008) Sorting of small RNAs into Arabidopsis argonaute complexes is directed by the 5′ terminal nucleotide. Cell 133: 116–127. 18342361 10.1016/j.cell.2008.02.034 PMC2981139

[40] Li B, Qin Y, Duan H, Yin W, Xia X (2011) Genome-wide characterization of new and drought stress responsive microRNAs in Populus euphratica. Journal of experimental botany 62: 3765–3779. 21511902 10.1093/jxb/err051 PMC3134338

[41] Zhang L, Chia J-M, Kumari S, Stein JC, Liu Z, et al (2009) A Genome-Wide Characterization of MicroRNA Genes in Maize. PLoS Genet 5: e1000716. 19936050 10.1371/journal.pgen.1000716 PMC2773440

[42] Nozawa M, Miura S, Nei M (2012) Origins and evolution of microRNA genes in plant species. Genome biology and evolution 4: 230–239. 22223755 10.1093/gbe/evs002 PMC3318440

[43] Cuperus JT, Fahlgren N, Carrington JC (2011) Evolution and Functional Diversification of MIRNA Genes. The Plant Cell 23: 431–442. 21317375 10.1105/tpc.110.082784 PMC3077775

[44] Cui H, Levesque MP, Vernoux T, Jung JW, Paquette AJ, et al (2007) An evolutionarily conserved mechanism delimiting SHR movement defines a single layer of endodermis in plants. Science 316: 421–425. 17446396 10.1126/science.1139531

[45] Ma Z, Coruh C, Axtell MJ (2010) Arabidopsis lyrata small RNAs: transient MIRNA and small interfering RNA loci within the Arabidopsis genus. The Plant cell 22: 1090–1103. 20407023 10.1105/tpc.110.073882 PMC2879747

[46] Fahlgren N, Howell MD, Kasschau KD, Chapman EJ, Sullivan CM, et al (2007) High-throughput sequencing of Arabidopsis microRNAs: evidence for frequent birth and death of MIRNA genes. PLoS ONE 2: e219. 17299599 10.1371/journal.pone.0000219 PMC1790633

[47] McClean PE, Mamidi S, McConnell M, Chikara S, Lee R (2010) Synteny mapping between common bean and soybean reveals extensive blocks of shared loci. BMC Genomics 11: 184. 20298570 10.1186/1471-2164-11-184 PMC2851600

[48] Lin JY, Stupar RM, Hans C, Hyten DL, Jackson SA (2010) Structural and functional divergence of a 1-Mb duplicated region in the soybean (Glycine max) genome and comparison to an orthologous region from Phaseolus vulgaris. Plant Cell 22: 2545–2561. 20729383 10.1105/tpc.110.074229 PMC2947175

[49] Allen E, Xie Z, Gustafson AM, Sung GH, Spatafora JW, et al (2004) Evolution of microRNA genes by inverted duplication of target gene sequences in Arabidopsis thaliana. Nat Genet 36: 1282–1290. 15565108 10.1038/ng1478

[50] Nozawa M, Miura S, Nei M (2012) Origins and Evolution of MicroRNA Genes in Plant Species. Genome Biol Evol 4: 230–239. 22223755 10.1093/gbe/evs002 PMC3318440

[51] Axtell MJ, Snyder JA, Bartel DP (2007) Common functions for diverse small RNAs of land plants. Plant Cell 19: 1750–1769. 17601824 10.1105/tpc.107.051706 PMC1955733

[52] Chen K, Rajewsky N (2007) The evolution of gene regulation by transcription factors and microRNAs. Nat Rev Genet 8: 93–103. 17230196 10.1038/nrg1990

[53] Li Y, Li C, Ding G, Jin Y (2011) Evolution of MIR159/319 microRNA genes and their post-transcriptional regulatory link to siRNA pathways. BMC evolutionary biology 11: 122. 21569383 10.1186/1471-2148-11-122 PMC3118147

[54] Millar AA, Gubler F (2005) The Arabidopsis GAMYB-like genes, MYB33 and MYB65, are microRNA-regulated genes that redundantly facilitate anther development. The Plant cell 17: 705–721. 15722475 10.1105/tpc.104.027920 PMC1069693

[55] Allen RS, Li J, Stahle MI, Dubroue A, Gubler F, et al (2007) Genetic analysis reveals functional redundancy and the major target genes of the Arabidopsis miR159 family. Proceedings of the National Academy of Sciences of the United States of America 104: 16371–16376. 17916625 10.1073/pnas.0707653104 PMC2042213

[56] Reyes JL, Chua NH (2007) ABA induction of miR159 controls transcript levels of two MYB factors during Arabidopsis seed germination. The Plant journal: for cell and molecular biology 49: 592–606. 17217461 10.1111/j.1365-313X.2006.02980.x

[57] Palatnik JF, Wollmann H, Schommer C, Schwab R, Boisbouvier J, et al (2007) Sequence and expression differences underlie functional specialization of Arabidopsis microRNAs miR159 and miR319. Dev Cell 13: 115–125. 17609114 10.1016/j.devcel.2007.04.012

[58] Ori N, Cohen AR, Etzioni A, Brand A, Yanai O, et al (2007) Regulation of LANCEOLATE by miR319 is required for compound-leaf development in tomato. Nature genetics 39: 787–791. 17486095 10.1038/ng2036

[59] Schommer C, Palatnik JF, Aggarwal P, Chetelat A, Cubas P, et al (2008) Control of jasmonate biosynthesis and senescence by miR319 targets. PLoS Biology 6: e230. 18816164 10.1371/journal.pbio.0060230 PMC2553836

[60] Thimm O, Bläsing O, Gibon Y, Nagel A, Meyer S, et al (2004) Mapman: a user-driven tool to display genomics data sets onto diagrams of metabolic pathways and other biological processes. Plant Journal 37: 914–939. 14996223 10.1111/j.1365-313x.2004.02016.x

[61] Chen K, An Y-Q (2006) Transcriptional Responses to Gibberellin and Abscisic Acid in Barley Aleurone. Journal of Integrative Plant Biology 48: 591–612.

[62] Langmead B, Trapnell C, Pop M, Salzberg SL (2009) Ultrafast and memory-efficient alignment of short DNA sequences to the human genome. Genome Biol 10: R25. 19261174 10.1186/gb-2009-10-3-r25 PMC2690996

[63] Zhang W, Zhou X, Xia J (2012) Identification of microRNAs and natural antisense transcript-originated endogenous siRNAs from small-RNA deep sequencing data. Methods in molecular biology 883: 221–227. 22589137 10.1007/978-1-61779-839-9_17

[64] Reese TA, Xia J, Johnson LS, Zhou X, Zhang W, et al (2010) Identification of novel microRNA-like molecules generated from herpesvirus and host tRNA transcripts. J Virol 84: 10344–10353. 20660200 10.1128/JVI.00707-10 PMC2937766

[65] Griffiths-Jones S, Saini HK, van Dongen S, Enright AJ (2008) miRBase: tools for microRNA genomics. Nucl Acids Res 36: D154–158. 17991681 10.1093/nar/gkm952 PMC2238936

[66] Hofacker IL, Fontana W, Stadler PF, Bonhoeffer LS, Tacker M, et al (1994) Fast folding and comparison of RNA secondary structures. Monatshefte für Chemie/Chemical Monthly 125: 167–188.

[67] Tamura K, Peterson D, Peterson N, Stecher G, Nei M, et al (2011) MEGA5: molecular evolutionary genetics analysis using maximum likelihood, evolutionary distance, and maximum parsimony methods. Molecular biology and evolution 28: 2731–2739. 21546353 10.1093/molbev/msr121 PMC3203626

[68] Saitou N, Nei M (1987) The neighbor-joining method: a new method for reconstructing phylogenetic trees. Molecular biology and evolution 4: 406–425. 3447015 10.1093/oxfordjournals.molbev.a040454

[69] Kimura M (1980) A simple method for estimating evolutionary rates of base substitutions through comparative studies of nucleotide sequences. Journal of Molecular Evolution 16: 111–120. 7463489 10.1007/BF01731581

[70] Lin JY, Stupar RM, Hans C, Hyten DL, Jackson SA (2010) Structural and functional divergence of a 1-Mb duplicated region in the soybean (Glycine max) genome and comparison to an orthologous region from Phaseolus vulgaris. The Plant cell 22: 2545–2561. 20729383 10.1105/tpc.110.074229 PMC2947175

