# Supplementary figures and images for: Systems and Evolutionary Characterization of MicroRNAs and Their Underlying Regulatory Networks in Soybean Cotyledons
Source: PLoS One. 2014 Jan 27;9(1):e86153. doi: 10.1371/journal.pone.0086153 (PMC3903507; doi:10.1371/journal.pone.0086153)

**average read counts**

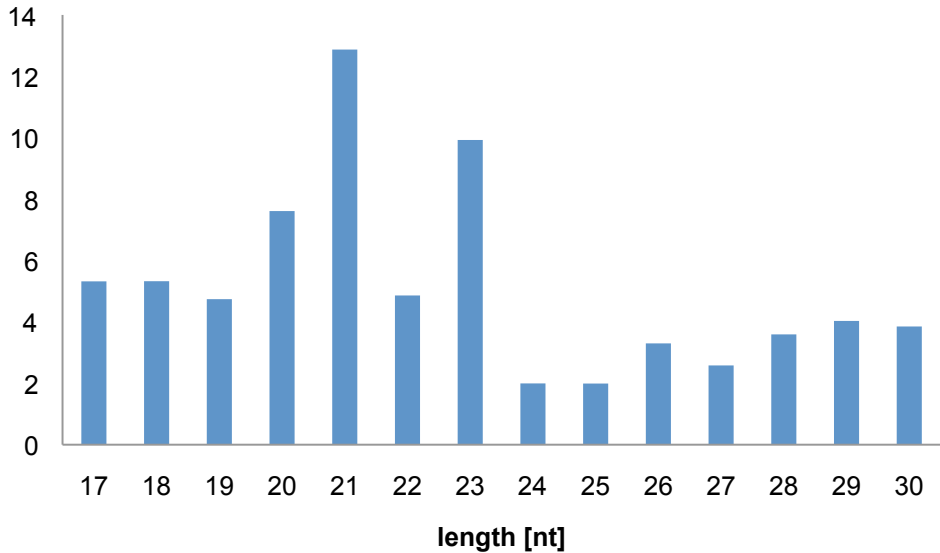

Supplement: Figure S1 — Average Accumulation Distribution of Small RNAs at Each Size in Soybean Cotyledons. The average count of reads per unique small RNA at each given size in cotyledon tissues is indicated on the Y-axis. The X-axis indicates the size of small RNAs. (PDF) [file pone.0086153.s001.pdf]

Inverted Repeats (IR)

soy-miRNA

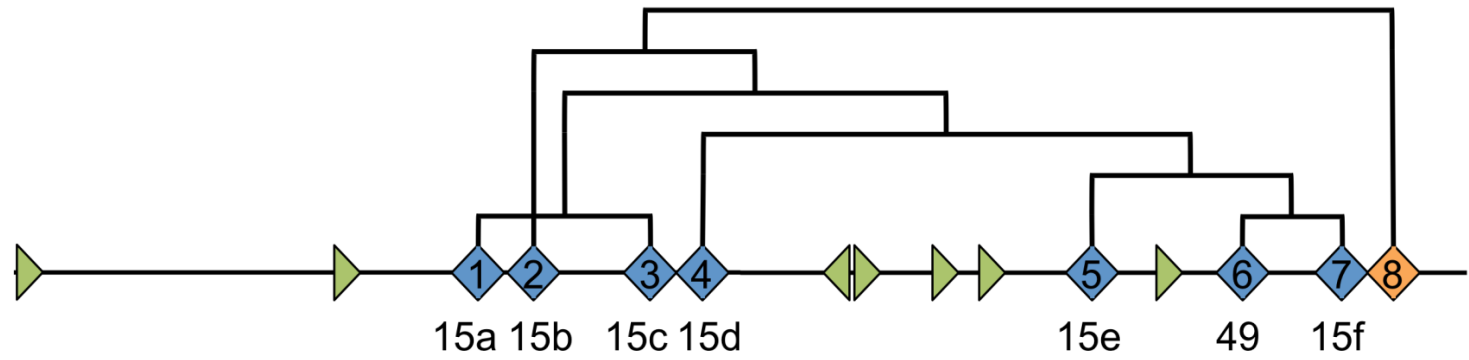

Genes

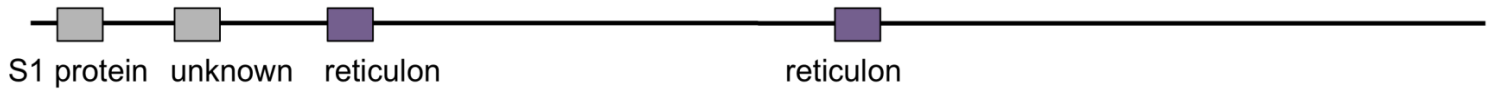

Direct Repeats

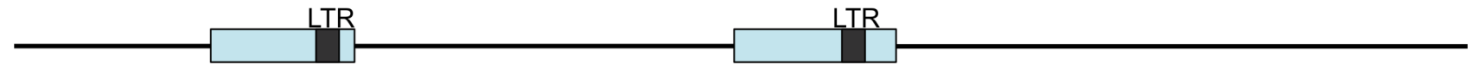

Supplement: Figure S2 — Soy-miR15/49 Gene Cluster Region. A 110 kb region on chromosome 3 contains multiple miRNA genes of the soy-miR15/49 family. Each of their miRNA fold-back structures are embedded in inverted repeat sequences (IR1-7) drawn as blue diamonds to represent their inverted structures. The inverted repeat sequence containing an indel in its middle region is illustrated as an orange diamond (IR8). The phylogenetic tree presented on the top indicates the evolutionarily relationship among those IRs. Truncated IRs homologous to the full-length IRs are shown as green triangles. Four genes (gray and purple rectangles) are located in this repetitive region. The reticulon protein genes are embedded in the 12 kb duplicated regions (light blue rectangle). The direct repeat region also contains a solo LTR (black rectangle). (PDF) [file pone.0086153.s002.pdf]

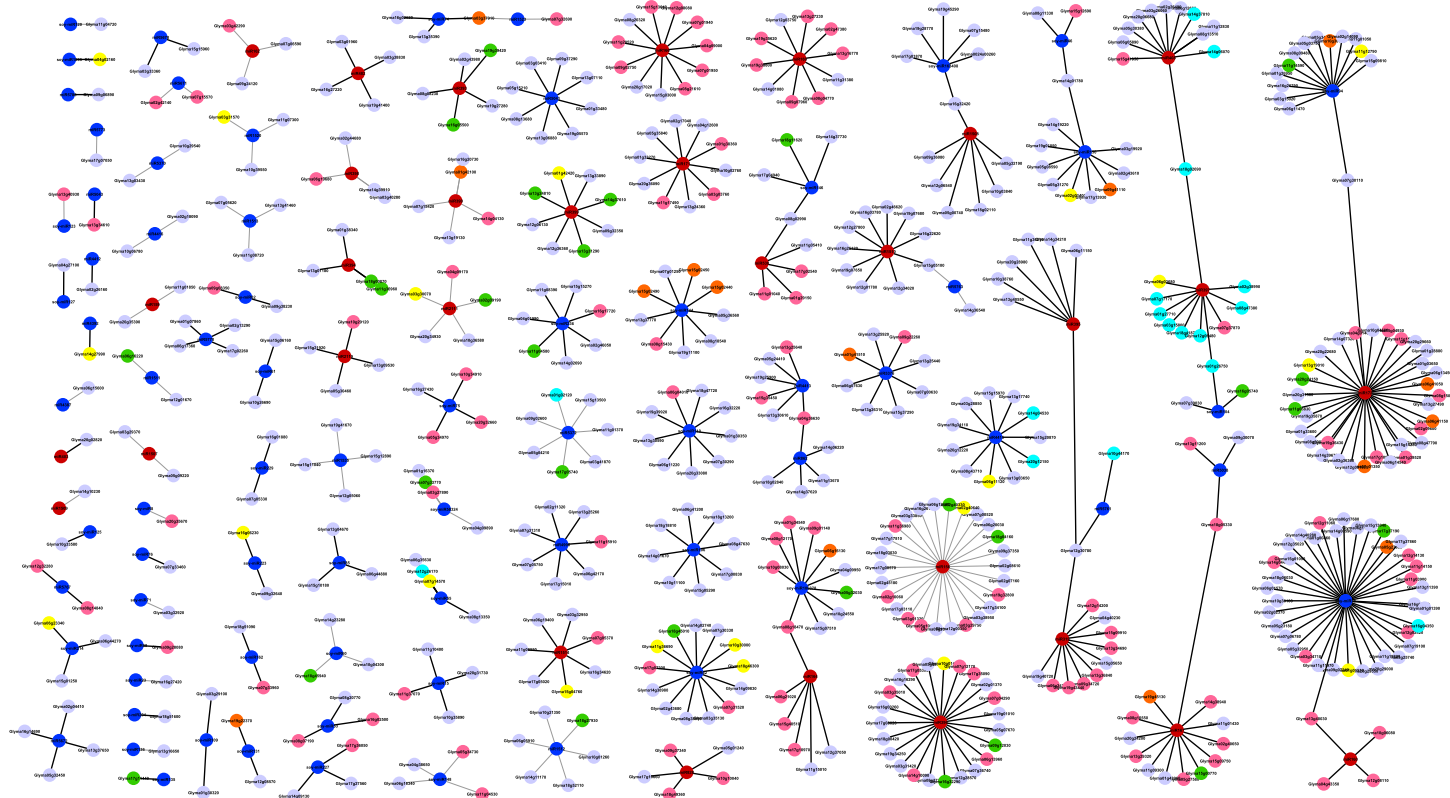

Supplement: Figure S3 — Global Topology of miRNA-target Networks. The conserved and non-conserved miRNAs are represented by red and blue circles, respectively. Target genes that were categorized into functional bins are shown. Targets encoding proteins related to RNA metabolism (pink circles), Ubiquitin based protein degradation (green circles), receptor kinase families (orange circles), secondary metabolism (turquoise circles), lipid metabolism (yellow circles) are distinguished from all other targets (light blue circles). Thick black edges connecting nodes indicate network overrepresented by cotyledon miRNA targets in preferentially regulated biological pathways. (PDF) [file pone.0086153.s003.pdf]

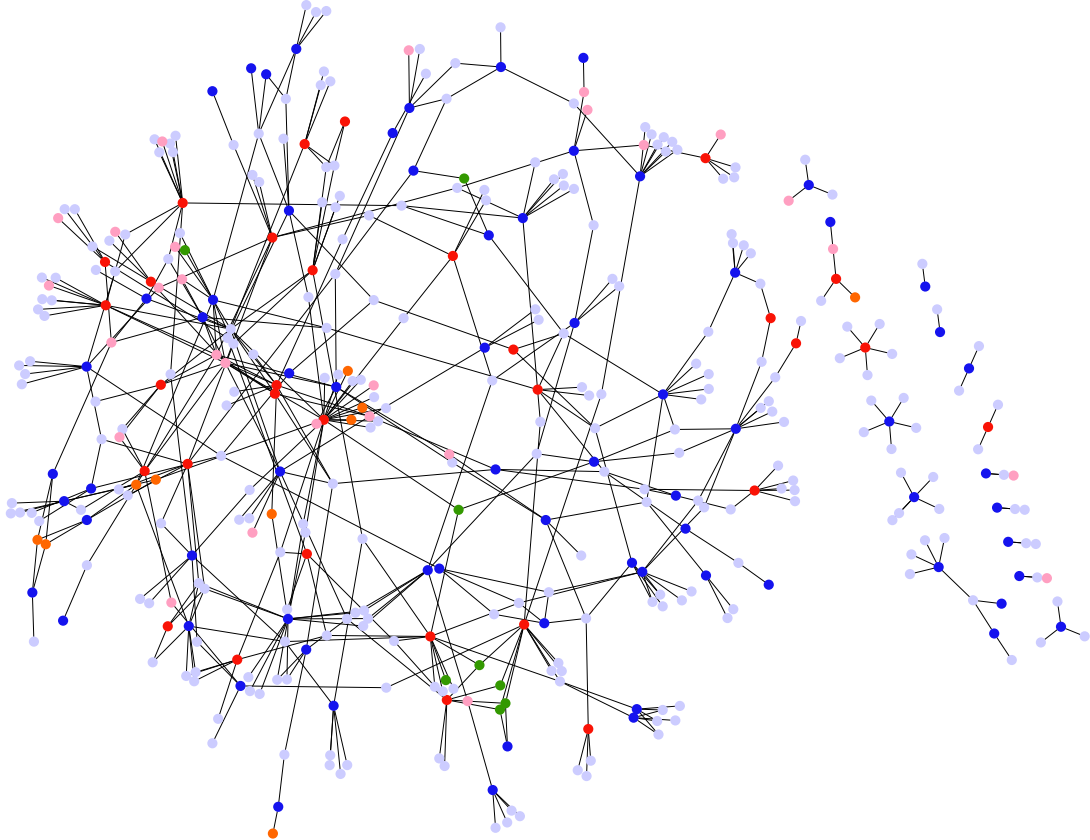

Supplement: Figure S4 — Global Topology of miRNA-Biological Pathway Networks. The conserved and non-conserved miRNAs were indicated by red and blue circles, respectively. Their targeted biological pathways are shown by pink circles representing biological pathways related to RNA metabolism, green circles standing for Ubiquitin based protein degradation pathways, orange circles denoting receptor kinase families and light blue circles representing all remaining pathways. (PDF) [file pone.0086153.s004.pdf]
